# A Rare Case of Advanced Urethral Diverticular Adenocarcinoma and a Review of Treatment Modalities

**DOI:** 10.1177/2324709619828408

**Published:** 2019-02-09

**Authors:** Fatai Oluyadi, Preethi Ramachandran, Vladmir Gotlieb

**Affiliations:** 1 Brookdale University Hospitals and Medical Center, New York, NY, USA

**Keywords:** urethral diverticular adenocarcinoma, adenocarcinoma, targeted therapy, urethral diverticulum

## Abstract

Female urethral diverticular cancer is a very rare entity with only around 100 cases reported so far in literature and accounts for <1% of all malignancies. In this article, we present a 47-year-old African American female with repeated hospital visits for urinary retention, hematuria, and urinary tract infections. Initial computed tomography imaging and cystoscopy was unremarkable except for a distended urinary bladder. Subsequent magnetic resonance imaging and corresponding cystoscopy eventually indicated the presence of a urethral diverticulum. She underwent urethral diverticulectomy and was found to have a mass arising from urethral diverticulum extending to vaginal walls. Her biopsy was suggestive of invasive adenocarcinoma in advanced stages, for which she subsequently underwent a total pelvic exenteration. Next-generation sequencing of the tumor showed CDKN2A/B loss, MSI-stable, and low TMB, thereby ruling out the options for targeted therapies. Extensive literature search and expert opinions were sought for her case since no consensus exists regarding the optimal therapeutic approach due to the rarity of this tumor. A final decision was made to treat her with platinum-based chemotherapy. Different treatment approaches including neoadjuvant chemoradiation followed by surgery, surgery followed by adjuvant chemotherapy, and surgery followed by chemoradiation have been tried. Platinum-based chemotherapy has generally been preferred based on an extensive literature search. Multimodality treatment approach seems to be the current approach to management for advanced stages for better overall survival. This case illustrates the challenges faced in making diagnosis and treatment decisions due to the rarity of this type of tumor and lack of consensus in the treatment approach.

## Introduction

The incidence of primary urethral cancer is estimated to be 4.3 per million in males and 1.5 per million in females in a study of roughly 10% of the US population,^1^ ultimately making up less than 1% of reported malignancies. The prevalence of urethral diverticula is about 1% to 6% in adult women with most exhibiting only benign features^2-4^ even making urethral diverticular malignancies rarer.

The earliest case of urethral diverticular carcinoma was reported in 1952.^5^ As of 1992, there were only a total of 59 cases reported in the English literature,^6^ and by 2009, there were about 76 cases reported.^7^ Most recent studies suggest that about 200 cases may have been reported thus far.^2^ The lack of established treatment strategies partly due to the few numbers of reported cases necessitates further study on the history and the evolution of the management for this very rare malignancy. We hope to highlight the various diagnostic and treatment modalities on the backdrop of the advances in the management of cancers today, particularly with the advent of targeted therapy. This report also aims to reiterate a simpler point of ruling out the possibility of malignant lesions in patients with urinary retention/obstruction especially in the lower urinary tract and should prompt thorough physical examination with palpation of the anterior vaginal wall and appropriate imaging.

## Case Presentation

This is the case of a 47-year-old female with medical history only remarkable for hypertension and asthma who first presented in November 2016 with urinary symptoms. She presented with hematuria and urinary frequency and was treated for a urinary tract infections with antibiotics. Subsequently, she continued to have urinary frequency, straining, and complete inability to void. She presented again in January 2017 with hematuria and urinary retention. Urology consult was obtained after staff encountered difficulty inserting a Foley catheter. The catheter was eventually inserted with a lot of resistance. Computed tomography (CT) scan done on the same visit was remarkable for a markedly distended bladder without evidence of obstruction by stone or evidence of hydronephrosis. She was scheduled for a urology clinic visit the following day where she was seen and instructed to remove the Foley catheter the day after. She returned to the emergency department after she removed the Foley catheter as instructed and was unable to urinate for up to 6 hours.

Cystoscopy done during multiple visits and magnetic resonance imaging evaluation eventually revealed the presence of periurethral cysts with a diagnosis of urethral diverticula prompting a urethral diverticulectomy. Biopsy results from samples taken during the diverticulectomy revealed an invasive adenocarcinoma. Follow-up cystoscopy did not show the ostium of the diverticulum but showed a friable mass that was adherent to the vaginal wall. CT scan of the chest, abdomen, and pelvis did not show any disease burden above the diaphragm. She did not have any evidence of bone metastasis during that presentation.

The patient underwent surgical resection of the mass with cystourethrectomy, anterior vaginectomy, total abdominal hysterectomy, bilateral salpingo-oophorectomy, bilateral pelvic lymph node dissection, appendectomy, a continent catheterizable reservoir (Indiana pouch) formation with primary ileocolonic re-anastomosis, and bilateral ureteral catheterization with a post-surgery diagnosis of T4N1MX stage 4 urethral diverticular adenocarcinoma. Margins post-surgery were found to be uninvolved with the adenocarcinoma.

Next-generation sequencing of tissue samples results showed CDKN2A/B loss, MSI-stable, TMB (tumor mutation burden)-low, and variants in the following genes: ATM, BARD1, FAT1, FLT4, KEAP1, MLL3, PIK3, and TAF1. Treatment with platinum-based regimens was decided based on extensive literature review and expert opinion and after genomic sequencing results showed no clear role of targeted therapy. A decision was made to treat her with chemotherapy combining platinum with 5-FU (fluorouracil), which has shown to improve progression-free survival when compared with other combination regimens. The patient has now received 5 cycles of cisplatin, 5-FU with leucovorin, and treatment has been well tolerated with no clinical signs of recurrence.

She continues to be followed by Urology and Oncology. Further need for concurrent radiotherapy was considered; however, the patient eventually refused.

## Discussion

As in other malignancies, the diagnosis of suspected urethral malignancies often includes a combination of CT/magnetic resonance imaging and endoscopic procedures (cystoscopy), aptly indicated in patients with suspected lesions obstructing urinary outflow with a classic collection of symptoms including urinary incontinence, irritative voiding symptoms, hematuria, recurrent urinary tract infection, and a palpable anterior vaginal/pelvic mass.^4,8-10^ In our case, suspicion of a mass arose with the difficult catheterization. After identifying these lesions, a multimodality therapeutic strategy including surgery, radiation, and chemotherapy is often indicated for definitive management.^9^ Multimodality therapy offers more benefit especially when the disease is advanced. Tissue diagnosis often following surgery usually highlights the nature of the disease. Adenocarcinomas, transitional- and squamous-type histology are the most commonly occurring, with the majority being adenocarcinoma.^7^ Cases of papillary urothelial carcinoma and papillary adenocarcinoma of the urethral diverticula were also reported.^11,12^ Further differentiation of the adenocarcinomatous types is seen, for example, in cases of clear cell and signet cell (a type of mucinous) adenocarcinoma.^2,13-16^ Of the 84 cases reviewed in this article, 64% were adenocarcinomas, 21% were transitional cell types, and 14% were squamous cell. The major prognostic factors for these types of lesions include the local extent of the tumor and the histologic characteristics, lymph node status, with some reports concluding that tumor size and histology were independent prognostic factors for survival and local tumor control.^17^ Another study reported that tumor stage, nodal status, and site of disease were independent predictors of survival.^18^ There is still a difference of opinion about the histologic types with the worst prognosis^6,17^; however, latest studies regarding chemotherapy and particularly targeted therapy are less focused on tumor histology, rather on the staging of the disease.^19^ There is also evidence that neoadjuvant chemotherapy with surgery offers better outcomes than surgery followed by adjuvant chemotherapy alone,^20^ and both these treatment plans involve platinum-based therapy as a common option for chemotherapy.

The adenocarcinoma of urethral diverticulum given its glandular origin is suspected to have arisen from the paraurethral glands as some studies have highlighted its staining properties with carcinoembryonic antigen.^21-23^ These lesions share similarities with other adenocarcinomas; hence, the suggestion of chemotherapy regimens such as platinum-based therapy like the 5-FU, leucovorin, and cisplatin combination are commonly used in colonic adenocarcinomas.^24,25^ Several other cancers especially those of genitourinary origins such as bladder cancer and primary urethral cancers are also known to have similar chemotherapeutic treatments as urethral diverticular cancers based on extrapolated evidence, hence the role of various other chemotherapeutic combinations that have been shown to have benefits, for example, gemcitabine/cisplatin (GC) combination and methotrexate/doxorubicin/vincristine/cisplatin (MVAC) combination.^26^ The difference in histologic types has also been seen to vary based on what part of the diverticular the tumor originates from and based on immunoreactivity with carcinoembryonic antigen and prostate-specific antigen.^21,22^

Before the use of chemotherapy for these tumors, most treatment plans involved surgery (eg, diverticulectomy, pelvic exenteration), radiation therapy alone, and surgery and radiation combined.^17^ The first example of multimodal therapy involving surgery, radiation, and chemotherapy was in 2003^10^ using cisplatin, 5-FU, and leucovorin. The treatment plan was also largely based on the stage of the disease, but it is important to note that patients often are diagnosed in the advanced stages of the disease.

We will now further examine the role of various treatment modalities including targeted therapy in the management of these urethral diverticular malignancies. Rare cases as this one gives us another opportunity to reexamine the evolution of cancer therapy and shine a light on how far we have come and the very long journey ahead.

### Surgery and Radiation

Historically, management of urethral lesions has involved surgery with a subsequent addition of radiation with or without surgery in some cases.^8^ A combination of surgery and radiation was studied showing a 44% recurrence with just diverticulectomy and/or radiation.^9^ Another study showed that the 5-year overall survival was 42%, and the 5-year cause-specific survival was 40% with aggressive treatment with surgery and radiotherapy.^17^ Aggressive treatment with surgery and radiation, albeit quite popular. is however not without a significant degree of complications.^17^ A more recent report shows a case of lymphatic disease progression 1 year after treatment with surgery and chemotherapy.^13^ Other reports, even more recent, show no recurrence at 12 months after being treated with surgery alone.^16,27^ It is important to note that these cases varied based on the stage, as less advanced stages were more amenable to surgery and radiation alone.

### Chemotherapy

Several studies reported benefits in patients treated with platinum-based therapy for urogenital malignancies including the urethral diverticular carcinoma.^10,20,26,28-30^ It is important here to note that some cancers may be platinum refractory, and proper identification of treatment options following a failure in platinum-refractory cases should be decided. The role of chemotherapy also varies whether it is used as adjuvant or neoadjuvant with or without radiation.^20^ Chemotherapy used as neoadjuvant with or without radiation has been shown to be superior to the adjuvant options.^20^ Commonly used platinum-based chemotherapy options include the 5-FU/leucovorin/cisplatin,^10^ GC,^26,29^ MVAC,^26^ and paclitaxel/ifosfamide/cisplatin^30^ with the only comparison between GC and MVAC showing benefits to similar degrees.^26^ GC or MVAC combinations may be used in patients with previous sensitivity to platinum-based therapy. Other taxane options (eg, docetaxel)^19^ also serve as an alternative therapy in patients with platinum-refractory cases. Irinotecan-based combinations reportedly show only a modest response as adjuvant regimens.^31^

### Chemoradiation

A good number of the studies we examined employed the use of chemotherapy in combination with radiation, and the first as mentioned earlier was in 2003^10^ mostly as neoadjuvant^20^ to shrink tumor size before surgery. Another report showed a case of a T4N0M0 primary urethral tumor treated with neoadjuvant paclitaxel, ifosfamide, and cisplatin combination followed by radiation with synchronous weekly cisplatin, and the patient was relapse-free 48 months after diagnosis, with normal voiding and sexual function.^30^

### When Platinum-Based Chemotherapy Fails

Treatment recommendations for patients who have failed platinum-based therapy include vinflunine^32,33^ and taxanes such as docetaxel although the latter is yet to be studied in a phase III trial but still believed to be a viable alternative as a salvage therapy.^19^ It is also important to note that patients who fail platinum-based therapy generally have poor prognosis.^33^ In another study,^34^ dramatic response was seen in a patient after treatment with nanoparticle albumin-bound paclitaxel after failure of ifosfamide/paclitaxel/cisplatin and irinotecan/5-FU/leucovorin combinations. For patients who have previously shown sensitivity to platinum-based therapy, the recommended combinations include the GC or the MVAC.

### Role of Targeted Therapy

The role of targeted therapy often comes into focus after patients have failed the initial platinum-based therapy. With the advent of PDL1 and PD1 based options such as atezolizumab, nivolumab, durvalumab, and pembrolizumab, and their use in wide variety of cancers, there is the opinion that urogenital cancers may also benefit from these new therapies. The KEYNOTE-045 trial examined 542 patients with advanced urothelial cancer that recurred or progressed after platinum-based chemotherapy and assigned them randomly to receive pembrolizumab versus investigator’s choice of chemotherapy with paclitaxel, docetaxel, or vinflunine. The pembrolizumab group showed a modest 3-month extension in overall survival compared with therapy with paclitaxel, docetaxel, or vinflunine with a lower rate of treatment-related adverse events.^33^ It is important to note that patients in the aforementioned study were selected irrespective of the PD1 or PDL1 expression, as the common criteria for selection were the disease stages and history of failure of platinum-based therapy, ultimately suggesting targeted therapy as a viable alternative when all else fails. A phase II trial is currently ongoing at the time of this report on the use of palbociclib, a CDK inhibitor, for metastatic urothelial cancer after failure of first-line chemotherapy; however, our patient could not be enrolled because genetic testing showed a loss in the CDK gene instead of an amplification. Options such as nivolumab combined with ipilimumab have shown benefits with cases of higher tumor mutational burden, but unfortunately not used in our patient as the TMB was low.^35^ On a final note, it is imperative that tissue diagnosis and treatment planning should include genomic sequencing to identify clearer roles for otherwise unmentioned targeted therapy options.

## Conclusion

Due to the paucity of information on urethral diverticular carcinomas, management of these cases can often be challenging. We, therefore, encourage prompt reporting of rare cases like this as it only further advances our understanding, thus allowing newer perspectives in management.

## Supplemental Material

Supplementary_information – Supplemental material for A Rare Case of Advanced Urethral Diverticular Adenocarcinoma and a Review of Treatment Modalities Click here for additional data file. Supplemental material, Supplementary_information for A Rare Case of Advanced Urethral Diverticular Adenocarcinoma and a Review of Treatment Modalities by Fatai Oluyadi, Preethi Ramachandran and Vladmir Gotlieb in Journal of Investigative Medicine High Impact Case Reports
