# Supplementary material for: A Rare Case of Advanced Urethral Diverticular Adenocarcinoma and a Review of Treatment Modalities
Source: J Investig Med High Impact Case Rep. 2019 Feb 9;7:2324709619828408. doi: 10.1177/2324709619828408 (PMC6376544; doi:10.1177/2324709619828408)
Supplement: Supplementary_information – Supplemental material for A Rare Case of Advanced Urethral Diverticular Adenocarcinoma and a Review of Treatment Modalities [file Supplementary_information.pdf]

Supplementary information:

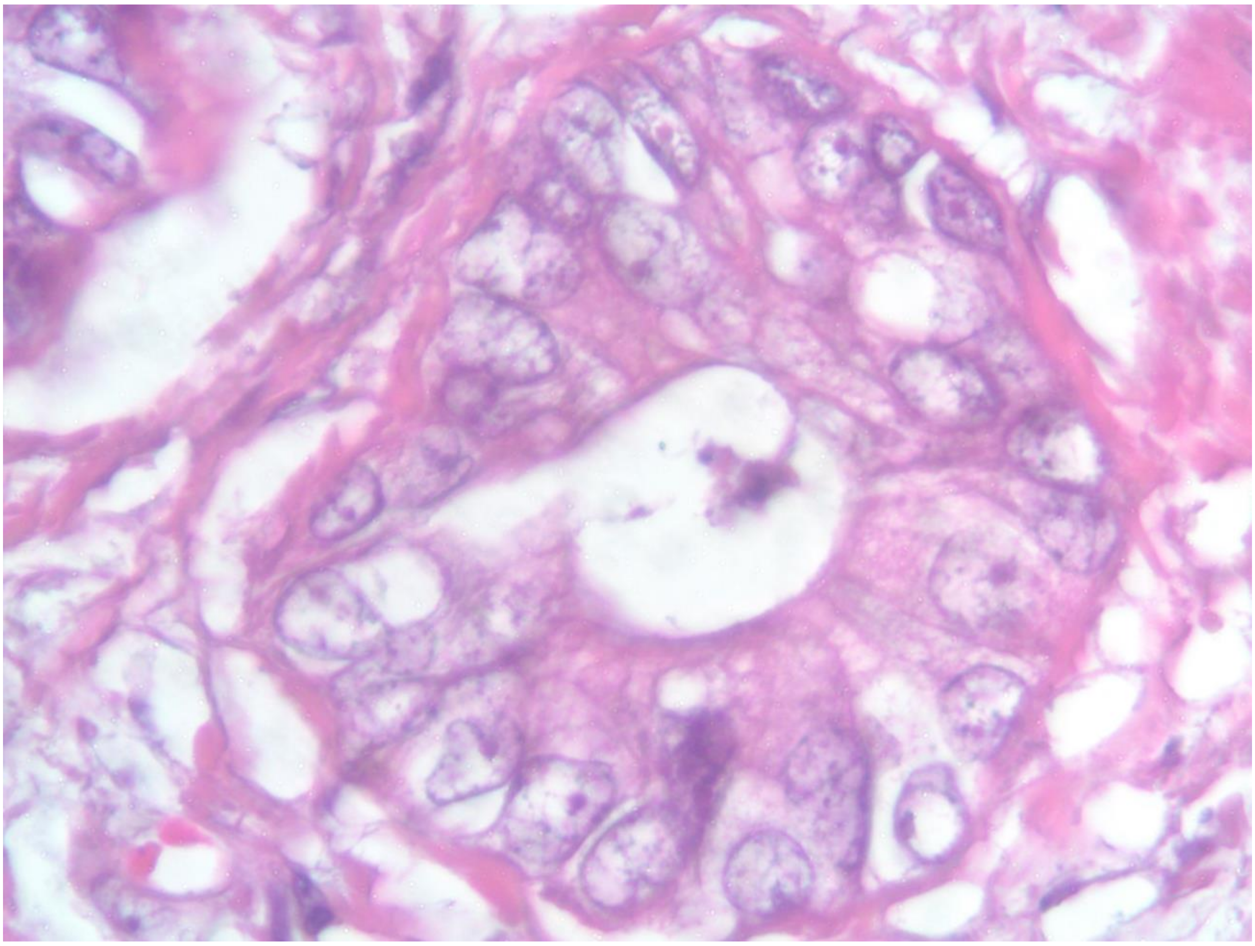

Figure 1: High Power field showing urethra diverticular adenocarcinoma comprised of cells with prominent nucleoli

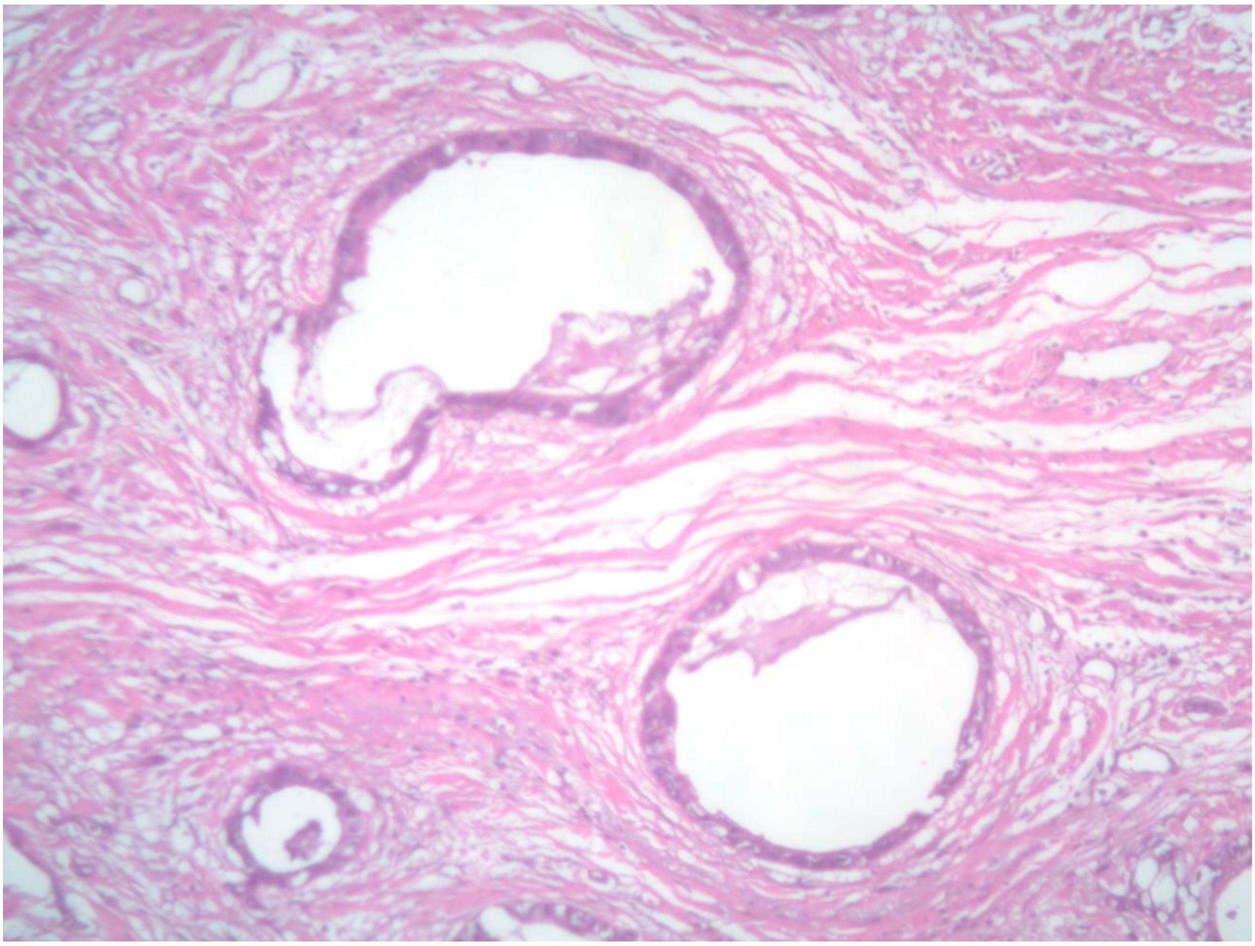

Figure 2: Image showing closer view of mucin in Lumen

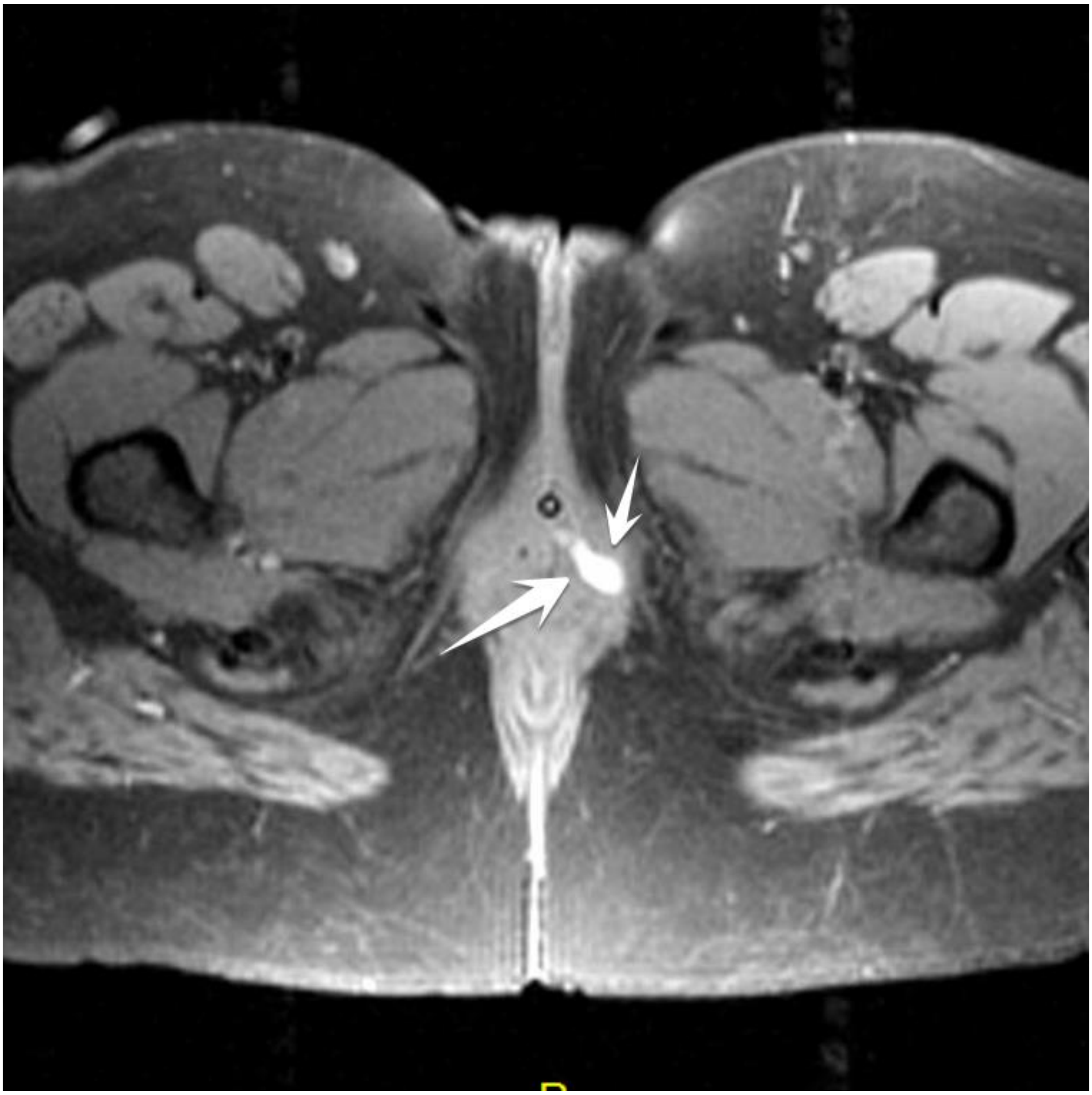

Figure 3: (Transverse View) MRI pelvis with contrast showing peri-urethral diverticula (White arrows)

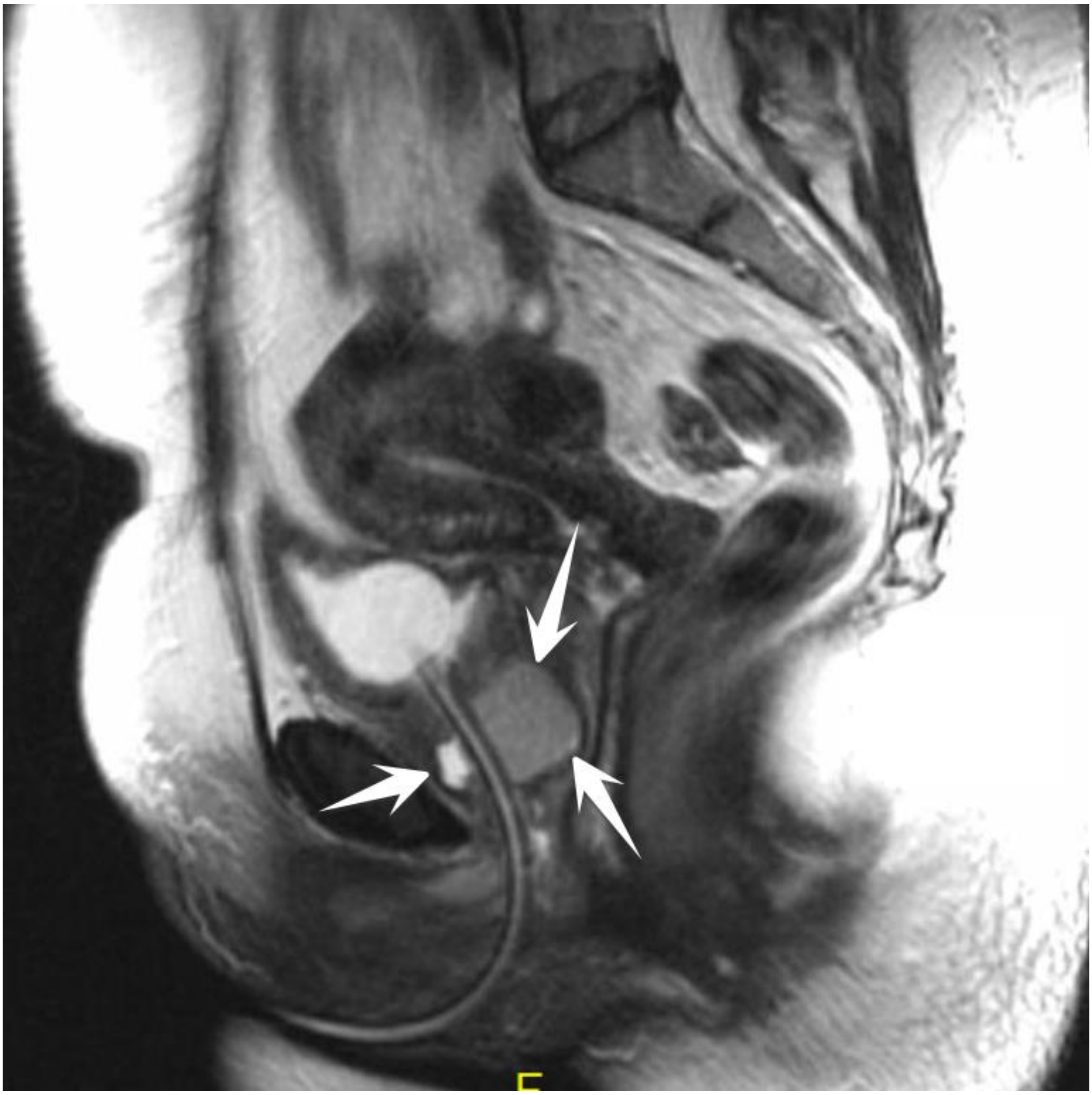

Figure 4: (Sagittal view) MRI pelvis with contrast showing peri-urethral diverticula (White arrows)

**Genomic Findings Detected**	**FDA-Approved Therapies (in patient's tumor type)**	**FDA-Approved Therapies (in another tumor type)**	**Potential Clinical Trials**

CDKN2A/B<br><br>(Loss)	None	None	None
Microsatellite status:<br><br>MS-Stable	None	None	None
Tumor Mutation<br><br>Burden:<br><br>TMB-Low;<br><br>5Muts/Mb	None	None	None

Table 1: Next Generation Sequencing Results; Note the CDKN loss which indicates the tumor is unlikely to be treated with Palbociclib. Ipilimumab and Nivolumab combinations have shown benefits in cases with high tumor mutation burden (TMB) however in this case the TMB is low.
